# Simultaneous Inhibition of Mcl-1 and Bcl-2 Induces Synergistic Cell Death in Hepatocellular Carcinoma

**DOI:** 10.3390/biomedicines11061666

**Published:** 2023-06-08

**Authors:** Marlen Michalski, Magdalena Bauer, Franziska Walz, Deniz Tümen, Philipp Heumann, Petra Stöckert, Manuela Gunckel, Claudia Kunst, Arne Kandulski, Stephan Schmid, Martina Müller, Karsten Gülow

**Affiliations:** Department of Internal Medicine I, Gastroenterology, Hepatology, Endocrinology, Rheumatology, and Infectious Diseases, University Hospital Regensburg Franz-Josef-Strauß-Allee 11, 93053 Regensburg, Germany; marlen.michalski@gmail.com (M.M.); magdalena.bauer@stud.uni-regensburg.de (M.B.); franziska1.walz@stud.uni-regensburg.de (F.W.); deniz.tuemen@ukr.de (D.T.); philipp.heumann@klinik.uni-regensburg.de (P.H.); petra.stoeckert@klinik.uni-regensburg.de (P.S.); manuela.gunckel@klinik.uni-regensburg.de (M.G.); claudia.kunst@ukr.de (C.K.); arne.kandulski@ukr.de (A.K.); stephan.schmid@ukr.de (S.S.); martina.mueller-schilling@ukr.de (M.M.)

**Keywords:** hepatocellular carcinoma (HCC), apoptosis, BH3-mimetics, ABT-199/venetoclax, MIK665/S64315

## Abstract

Despite the recent approval of new therapies, the prognosis for patients with hepatocellular carcinoma (HCC) remains poor. There is a clinical need for new highly effective therapeutic options. Here, we present a combined application of BH3-mimetics as a potential new treatment option for HCC. BH3-mimetics inhibit anti-apoptotic proteins of the BCL-2 family and, thus, trigger the intrinsic apoptosis pathway. Anti-apoptotic BCL-2 proteins such as Bcl-2 and Mcl-1 are frequently overexpressed in HCC. Therefore, we analyzed the efficacy of the two BH3-mimetics ABT-199 (Bcl-2 inhibitor) and MIK665 (Mcl-1 inhibitor) in HCC cell lines with differential expression levels of endogenous Bcl-2 and Mcl-1. While administration of one BH3-mimetic alone did not substantially trigger cell death, the combination of two inhibitors enhanced induction of the intrinsic apoptosis pathway. Both drugs acted synergistically, highlighting the effectivity of this specific BH3-mimetic combination, particularly in HCC cell lines. These results indicate the potential of combining inhibitors of the BCL-2 family as new therapeutic options in HCC.

## 1. Introduction

One of the hallmarks of cancer pathophysiology is resistance toward apoptosis [1]. Expressions of anti-apoptotic and pro-apoptotic BCL-2 family members, which control the intrinsic apoptosis pathway, are often altered in cancer. This correlates with resistance to therapy. The BCL-2 family consists of three groups of structurally related proteins: anti-apoptotic Bcl-2-like proteins (Bcl-2, Bcl-X_L_, Bcl-W, Mcl-1, A1/BFL-1 and Bcl-B), pro-apoptotic Bax/Bak proteins with multiple BH-domains (Bax, Bak and Bok), and pro-apoptotic BH3-only proteins (Bim, Puma, Bid, NOXA, Bid, Bmf, Bik, and HRK).

Upon initiation of intrinsic apoptosis, BH3-only proteins are upregulated. They inhibit anti-apoptotic BCL-2 family members such as Bcl-2 and Mcl-1, activating Bax and Bak. Activation of Bax and Bak results in mitochondrial outer membrane permeabilization (MOMP). MOMP promotes the release of cytochrome c from mitochondria [2,3]. Cytochrome c stimulates the oligomerization of APAF-1, which recruits pro-caspase-9 to form the apoptosome, enabling auto-cleavage and activation of caspase-9. Activated caspase-9 cleaves the effector caspases-3 and -7. These effector caspases cause irreversible induction of apoptosis [2]. Due to their important function in the BCL-2-regulated apoptosis pathway, BH3-only proteins are crucial for determining whether the fate of the cell shifts toward survival or death [4]. Therefore, many anticancer drugs aim to induce cell death in tumor cells via indirect activation of BH3-only proteins by modulation of p53 or glucocorticoid receptor pathways [5,6]. However, in tumor tissue, proteins within these upstream signaling cascades are often mutated, deleted, or their expression is epigenetically suppressed during tumor development [7]. These cellular adaptations often result in resistance to chemotherapeutics. To bypass resistance, a new class of therapeutics, called BH3-mimetics, has been developed [3,8]. BH3-mimetics imitate the effect of BH3-only proteins. They inhibit anti-apoptotic BCL-2 proteins and therefore induce apoptosis.

The first clinically approved BH3-mimetic is the selective Bcl-2 inhibitor ABT-199/venetoclax [9]. It was approved by the Food and Drug Administration (FDA, USA) and the European Medicines Agency (EMA) in 2016 for the treatment of chronic lymphocytic leukemia (CLL) [10,11]. Resistance or reduced response to ABT-199 has been reported to be associated with the overexpression of anti-apoptotic molecules such as Bcl-X_L_ and/or Mcl-1 [12]. Other resistant tumors rely on increased expression of multiple anti-apoptotic BCL-2-molecules for survival, and therefore do not respond to monotherapy with ABT-199 in the first place [13]. ABT-199 has been combined with various drugs to improve efficacy and overcome resistance toward cell death. It is already approved in combination with obinutuzumab or rituximab in CLL and azacitidine, decitabine, or cytarabine in acute myeloid leukemia (AML) [14]. Several recent clinical trials and in vivo studies use ABT-199 in combination with other pharmaceuticals in different tumor entities [3,15]**,** including solid tumors such as breast and pancreatic cancer, as well as hematopoietic neoplasia [16,17].

MIK665/S64315 is a BH3-mimetic specifically inhibiting Mcl-1. It has achieved considerable effects in mouse models of solid and hematologic cancer entities, while sparing healthy tissue [18]. In addition, Mcl-1 inhibitors are currently being investigated in clinical trials for various hematologic malignancies [15]. As Mcl-1 plays a major role in resistance to ABT-199 and other chemotherapeutics, combining ABT-199 and MIK665 is a promising approach to improve therapeutic efficacy [12,19].

Hepatocellular carcinoma (HCC) is the most frequent liver tumor entity and the third-leading cause of cancer-related death worldwide [20]. Barcelona Clinic Liver Cancer (BCLC) publishes treatment algorithms depending on the tumor stage. In 2022, the updated BCLC algorithm redefined the intermediate-stage HCC (BCLC-B) and the treatment sequence for systemic treatment has been addressed in detail [21]. Systemic treatment is recommended for patients with compensated liver function and HCC in the intermediate stage (BCLC-B) that cannot be treated locally, and those in advanced stage with multifocal disease or distant metastasis (BCLC-C). Combination therapies with immunotherapeutic agents are effective and superior to tyrosine kinase inhibitors, such as sorafenib or lenvatinib [22]. The combination of atezolizumab (neutralizing humanized monoclonal anti-PD-L1 antibody) and bevacizumab (neutralizing humanized monoclonal anti-VEGF antibody) was approved by the FDA and EMA in 2020 and is now an established first-line treatment option. Very recently, data from the HIMALAYA-trial (NCT03298451) led to the approval of the combination of tremelimumab (neutralizing humanized monoclonal anti-CTLA-4 antibody) and durvalumab (neutralizing humanized monoclonal anti-PD-L1 antibody). Other combination therapies, such as ipilimumab (anti-CTLA antibody) plus nivolumab (anti-PD-L1 antibody), are still under investigation in current recruiting phase III clinical studies (NCT04039607 (CheckMate-9DW)). Patients with contraindications for immunotherapy should be treated with tyrosine kinase inhibitors lenvatinib or sorafenib.

After progression under first-line treatment, the tyrosine kinase inhibitors regorafenib (for patients who initially responded to sorafenib) and cabozantinib are possible treatment options in the second-line setting. For patients with high expression of alpha-feto-protein (AFP > 400 ng/mL), ramucirumab (neutralizing humanized anti-VEGFR2 antibody) is recommended [22,23,24,25,26,27]. Reliable, current data and clinical evidence for an optimal treatment sequence after progression under immunotherapy are rare. HCC remains a highly therapy-resistant tumor entity and, therefore, challenging to treat [24].

Thus, it is important to identify new therapeutic targets for HCC. In addition, markers to predict sensitivity and resistance to certain treatments are highly important. In HCC, the expression of pro- and anti-apoptotic BCL-2 molecules is often altered. In particular, Mcl-1 is overexpressed and functions as a crucial survival factor in HCC [28,29,30,31,32]. There are other anti-apoptotic BCL-2 family members overexpressed in tumors. Consistent with these findings, inhibition of Bcl-2 increased the chemosensitivity of HCC cell lines [33,34]. Using the BH3-mimetic ABT-737, which inhibits Bcl-2, Bcl-X_L_ and Bcl-W, induced apoptosis in HCC when Mcl-1 expression was simultaneously inhibited, e.g., by sorafenib [35]. In addition, treatment with the Bcl-X_L_ inhibitor A-1331852 restores sensitivity to HepG2 cells resistant to regorafenib [36]. ABT-737 and the pan-Bcl-2 inhibitor obatoclax reduce cell migration and adhesion in HCC [37]. These studies demonstrate that BH3-mimetics have a high potential in treating HCC. However, the efficacy of BH3-mimetics specifically for Mcl-1 in combination with other BCL-2 inhibitors has not been addressed in HCC.

This study analyzed the therapeutic potential of the Mcl-1 inhibitor MIK665 and the Bcl-2 inhibitor ABT-199 in different HCC cell lines. Both drugs were tested either alone or in combination.

## 2. Materials and Methods

### 2.1. Chemicals

ABT-199, MIK665, carbonyl cyanide-p-trifluoromethoxyphenylhydrazone (FCCP), and zVAD were obtained from Selleck Chemicals GmbH, Planegg, Germany. Tetramethylrhodamine ethyl ester (TMRE) was obtained from Invitrogen, Waltham, MA, USA. Cells were treated with ABT-199, MIK665, or both in the given concentrations. Co-treatment with zVAD (35 µM) was performed 20 min prior to treatment with ABT-199 and MIK665. Cells were incubated for the indicated time.

### 2.2. Cells

Hep3B cells were cultured in MEM, Huh7 cells in DMEM (high glucose) and HepG2 cells in RPMI-1640 medium. All media were supplemented with 10% FCS. Hep3B (Hep3B 2.1-7 [ATCC HB-8064]) and HepG2 (HepG2 [ATCC HB-8065]) were obtained from American Type Culture Collection (ATCC, Manassas, VA, USA) and Huh7 (CLS 300156) from Cell Lines Service (CLS, Eppelheim, Germany).

### 2.3. Cell Death Assays

Cell death was assessed 24 h and 48 h after administering specific treatments consisting of ABT-199, MIK665, or both. Cells were harvested and stained with Annexin V-FITC and DAPI (Becton Dickinson (BD) Biosciences, Heidelberg, Germany) and were analyzed using flow cytometry (LSRFortessa^TM^ Cell Analyzer, BD Biosciences, Heidelberg, Germany). To compare results between experiments, a normalized value (specific death [%]) was calculated as follows [38,39,40]:

$$specific\;cell\;death\left[\%\right]=\frac{\left(experimental\;cell\;death\left[\%\right]-spontaneous\;\left(background\right)\;cell\;death\left[\%\right]\right)}{\left(100-spontaneous\;\left(background\right)\;cell\;death\left[\%\right]\right)}\times 100.$$

### 2.4. Luciferase Activity Assays

Caspase activity was determined using Caspase-Glo assay systems (Promega, Madison, WI, USA). Luminescent signals were detected using a TriStar2 LB 942 Multimode Reader (Berthold Technologies, Bad Wildbad, Germany). Specific increase in caspase activity was calculated using the following formula [41]:

$$specific\;increase\;in\;activity\;\left[\%\right]=\frac{{LI}_{treated}-{LI}_{control}}{{LI}_{control}}\times 100$$

LI = luminous intensity.

### 2.5. Immunofluorescence

Cells were fixed in 4% paraformaldehyde (Alfa Aesar Chemicals, Haverhill, MA, USA) and permeabilized in 0.1% Triton X-100 (Sigma-Aldrich, St. Louis, MI, USA). Antibodies against cytochrome c (ab110325, Abcam, Cambridge, UK) were diluted 1:500, antibodies against TOMM20 (42406, Cell Signaling Technology, Danvers, MA, USA) were diluted 1:100. Secondary antibodies against rabbit IgG (A-11008, Invitrogen, Waltham, MA, USA) and mouse IgG (A-11005, Invitrogen, Waltham, MA, USA) were diluted 1:1000. Specific staining was analyzed using fluorescence microscopy (Keyence BZ-X810, Keyence, Osaka, Japan). The contrast and brightness of the single stainings were adjusted in the individual fluorescence images to optimize the detection of a possible cytochrome c release.

### 2.6. Western Blot

Western blot analysis was performed as described previously [42]. Antibodies against full-length and cleaved caspase-9 (PA5-17913, Invitrogen, Waltham, Massachusetts, USA), full-length and cleaved caspase-3 (9662, Cell Signaling Technology, Danvers, MA, USA), full-length and cleaved PARP (9542, Cell Signaling Technology, Danvers, MA, USA), Mcl-1 (4572, Cell Signaling Technology, Danvers, MA, USA), Bcl-2 (ab182858, Abcam, Cambridge, UK), Bcl-X_L_ (2762S, Cell Signaling Technology, Danvers, MA, USA), p53 (sc-126, Santa Cruz Biotechnology, Dallas, Texas, USA ) and NOXA (ab13654, Abcam, Cambridge, UK) were diluted 1:1000. Secondary antibodies against rabbit IgG (A0545, Sigma-Aldrich, St. Louis, MI, USA) were diluted 1:10,000 and secondary antibodies against mouse IgG (A9044, Sigma Aldrich, St. Louis, MI, USA) were diluted 1:10,000.

### 2.7. Graphics and Calculations

All graphs and statistical analyses were created with GraphPad Prism 8 Software (Graphpad Software, Inc., San Diego, CA, USA). Significances were determined using unpaired *t*-tests. The Combination Index was calculated with the CompuSyn software (http://www.combosyn.com/ (accessed on 26 May 2023)) Version 1 [43,44]. Schematics were created with BioRender (https://biorender.com/ (accessed on 26 May 2023)).

## 3. Results

### 3.1. HCC Cell Lines Display Different Expression Levels of Bcl-2 and Mcl-1

**Figure 1:**
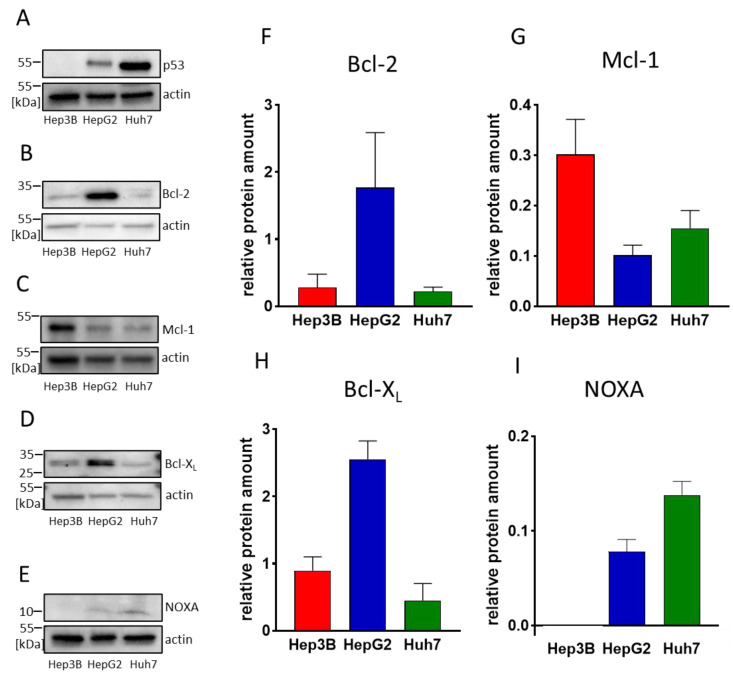
Determination of p53 and BCL-2 family protein expression in different HCC cell lines. (**A**–**E**) Western blot analyses of p53 and BCL-2 family members Bcl-2, Mcl-1, Bcl-X_L_ and NOXA in Hep3B, HepG2 and Huh7. The three cell lines displayed a differential expression pattern. Shown is a representative western blot. (**F**–**I**) Densitometric analysis of western blots: Shown are the relative amounts of Bcl-2, Mcl-1, Bcl-X_L_ and NOXA in Hep3B (red), HepG2 (blue) and Huh7 (green) normalized to β-actin (*n* = 3, mean ± SD).

In tumor cells, Bcl-2 and Mcl-1 are often overexpressed. This leads to increased resistance toward apoptosis and to dependence on these survival factors [45]. Inhibition of anti-apoptotic BCL-2 molecules by BH3-mimetics, such as ABT-199/venetoclax and MIK665/S64315, can therefore lead to induction of apoptosis. To investigate whether the HCC cell lines Hep3B, HepG2, and Huh7 represent a model system to analyze the potential of ABT-199 and MIK665 for HCC treatment, we examined the protein expression of Bcl-2 and Mcl-1 in these cell lines. To include the role of p53, which is an important upstream regulator of BH3-only proteins, we ensured the differential p53 status for the cell lines being used (p53 is deleted in Hep3B, wild-type p53 is expressed in HepG2, and mutant p53 is present in Huh7) (Figure 1A).

HepG2 showed the highest protein expression of Bcl-2 (Figure 1B,F) while Hep3B revealed the highest expression of Mcl-1 (Figure 1C,G). Huh7 displayed low levels of both proteins (Figure 1B,C,F,G). Since other BCL-2 family members may also have an impact on BH3-mimetics-mediated induction of cell death, we examined the expression of Bcl-X_L_ (Figure 1D,H) and NOXA (Figure 1E,I). It is known that a high Bcl-X_L_ expression can compensate for the inhibition of Bcl-2 [32,46], while NOXA is known to enhance the degradation of Mcl-1 and thus may enhance effects induced by Mcl-1 inhibitors [47]. HepG2 showed the highest expression of Bcl-X_L_, whereas Hep3B and Huh7 displayed a moderate Bcl-X_L_ expression. In Hep3B cells, we could not detect NOXA expression. HepG2 and Huh7 showed moderate NOXA expression (Figure 1D,E,H,F).

### 3.2. Combination of BH3-Mimetics Induces Release of Cytochrome c into the Cytosol

**Figure 2:**
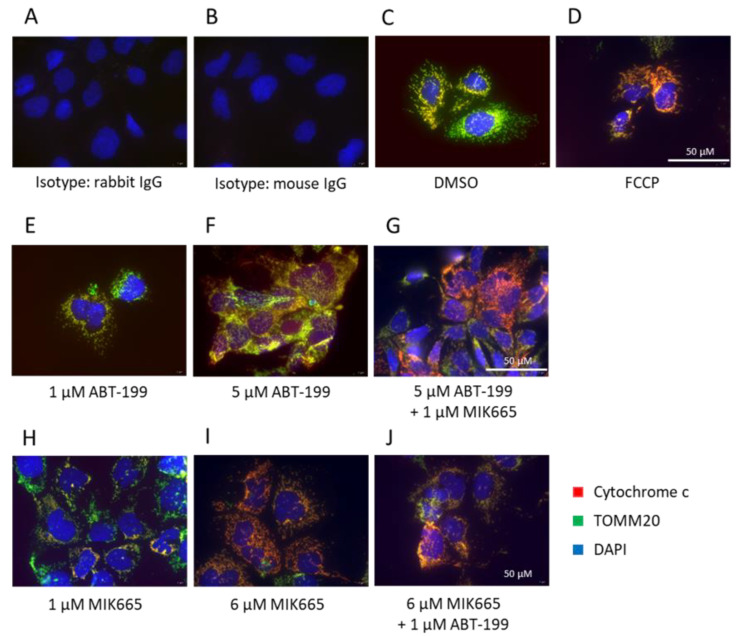
Combination of ABT-199 and MIK665 results in cytochrome c release in Hep3B cells. (**A**,**B**) Corresponding isotype controls. (**C**) DMSO treatment was used as vehicle control. (**D**) Cells were treated with 30 µM FCCP for 3 h to induce MOMP as a positive control. (**E**–**J**) Immunofluorescence of TOMM20 and cytochrome c in Hep3B cells. Cells were subjected to ABT-199 and MIK665 for 4 h at the indicated concentrations as single or combined treatment (*n* = 3).

BH3-mimetics inhibit anti-apoptotic members of the BCL-2 family, resulting in the release of cytochrome c from mitochondria into the cytosol, which is the initial step of the intrinsic apoptosis pathway. However, the anti-apoptotic BCL-2 proteins can substitute for each other [48]. To evaluate whether a single or combined treatment with ABT-199 and MIK665 can initiate intrinsic apoptosis in HCC, we examined the induction of MOMP and cytochrome c release upon treatment with ABT-199 and MIK665 in Hep3B cells. MOMP was determined using flow cytometry with TMRE staining. TMRE is a cell-permeable, cationic dye that accumulates in the mitochondrial matrix of active mitochondria with intact mitochondrial membrane potential. Induction of MOMP results in reduced membrane potential, and, therefore, reduced TMRE staining. The mitochondrial uncoupler carbonyl cyanide-p-trifluoromethoxyphenylhydrazone (FCCP) was used as a positive control for membrane depolarization (Supplementary Figure S1A). Application of 5 µM ABT-199 did not cause depolarization of the mitochondria. After application of 6 µM MIK665, some cells showed a modest loss of membrane potential. Similar results were seen for a combination of 1 µM ABT-199 and 6 µM MIK665. The combination of 5 µM ABT-199 and 1 µM MIK665 resulted in a clear induction of MOMP (Supplementary Figure S1B–E). Cytochrome c release was analyzed using immunofluorescence. Translocase of outer mitochondrial membrane 20 (TOMM20) was used as a marker for mitochondria. FCCP was applied as a positive control for the induction of cytochrome c release (Figure 2A–D, Supplementary Figure S2A,B). Application of a single BH3-mimetic induced only moderate or no cytochrome c release. Consistent with the data on the induction of MOMP (Supplementary Figure S1), only the highest dose (6 µM) of MIK665 showed a moderate release of cytochrome c in Hep3B cells (Figure 2I, Supplementary Figure S2G). Lower concentrations of MIK665 or single treatment with ABT-199 did not result in cytochrome c release (Figure 2E,F,H, Supplementary Figure S2C,D,F). Thereafter, we tested whether simultaneous inhibition of Bcl-2 and Mcl-1 by ABT-199 and MIK665 can induce cytochrome c release. The combination of 5 µM ABT-199 and 1 µM MIK665, as well as the combination of 6 µM MIK665 and 1 µM ABT-199, led to the release of cytochrome c after 4 h of treatment (Figure 2G,J, Supplementary Figure S2E,H). This confirms the results obtained from measuring the mitochondrial membrane potential (Supplementary Figure S1). Thus, the combined inhibition of two members of the BCL-2 family in HCC cells cannot be compensated by cellular resistance mechanisms.

### 3.3. Combination Treatment of ABT-199 and MIK665 Induces a Caspase Cascade

**Figure 3:**
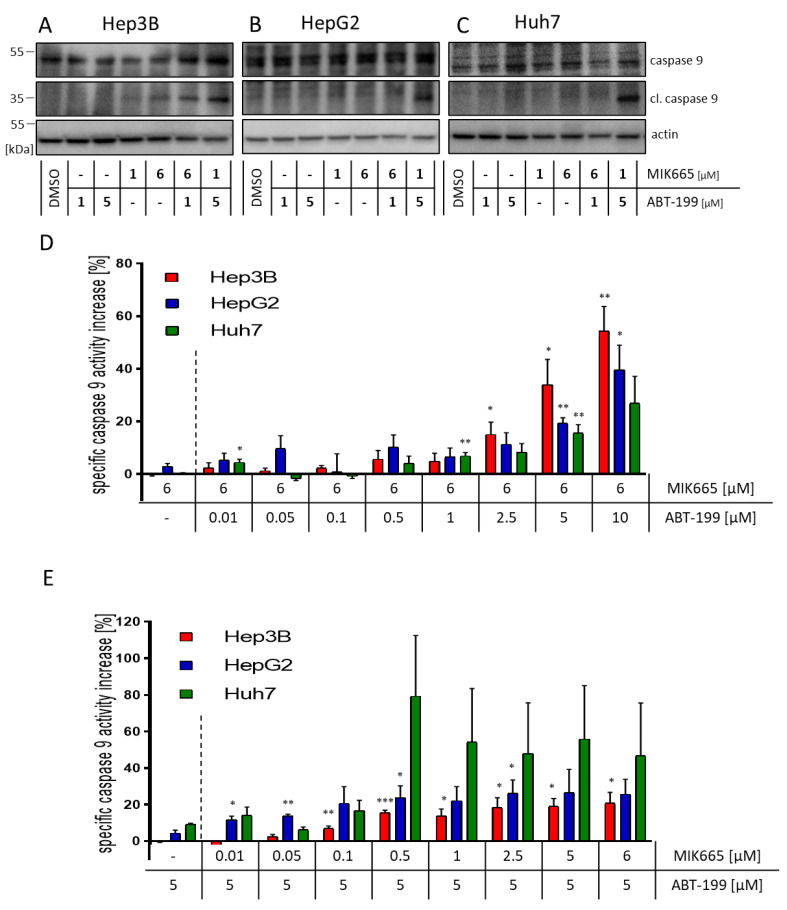
Combination of the BH3-mimetics ABT-199 and MIK665 results in increased cleavage and activity of caspase-9 in HCC cell lines. (**A**–**C**) Western blot analyses of (cleaved) caspase-9. Shown is a representative western blot. Cells were treated with either one BH3-mimetic or a combination of ABT-199 or MIK665 inhibitors. DMSO was used as a vehicle control. (**D**,**E**) Activity assays of caspase-9 in HCC cell lines (Hep3B (red), HepG2 (blue), and Huh7 (green)) after 4 h of treatment with either ABT-199 or MIK665 alone or in combination at the indicated concentrations (the dotted line separates the single treatment from the combination treatment with ABT-199 and MIK665). Data show a specific increase in activity compared to DMSO-treated controls (*n* = 3, mean ± SEM) (* *p* < 0.05; ** *p* < 0.01; *** *p* < 0.001 compared to treatment with one inhibitor alone).

Release of cytochrome c leads to activation of initiator caspase-9. As expected, we demonstrated that one individual inhibitor could not induce cleavage of caspase-9 in the HCC cell lines HepG2 and Huh7 (Figure 3B,C). Only in Hep3B cells, a moderate cleavage upon treatment with 1 µM MIK665 and 6 µM MIK665 was observed (Figure 3A). In contrast, the combined administration of 5 µM ABT-199 and 1 µM MIK665 resulted in caspase-9 cleavage after 4 h in all cell lines (Figure 3A–C). In addition to caspase cleavage, the combination treatment resulted in increased caspase-9 activity in the HCC cell lines after 4 h (Figure 3D,E). Thus, caspase-9 activity was markedly enhanced after combination treatment. Levels of caspase-9 activity remained constant for up to 6 h of treatment (Supplementary Figure S3A–F). Caspase-9 is activated in all cell lines irrespective of their p53 status or NOXA expression. The p53 status and NOXA expression therefore do not seem to markedly influence response to ABT-199 and MIK665 (Figure 1A,E,I).

**Figure 4:**
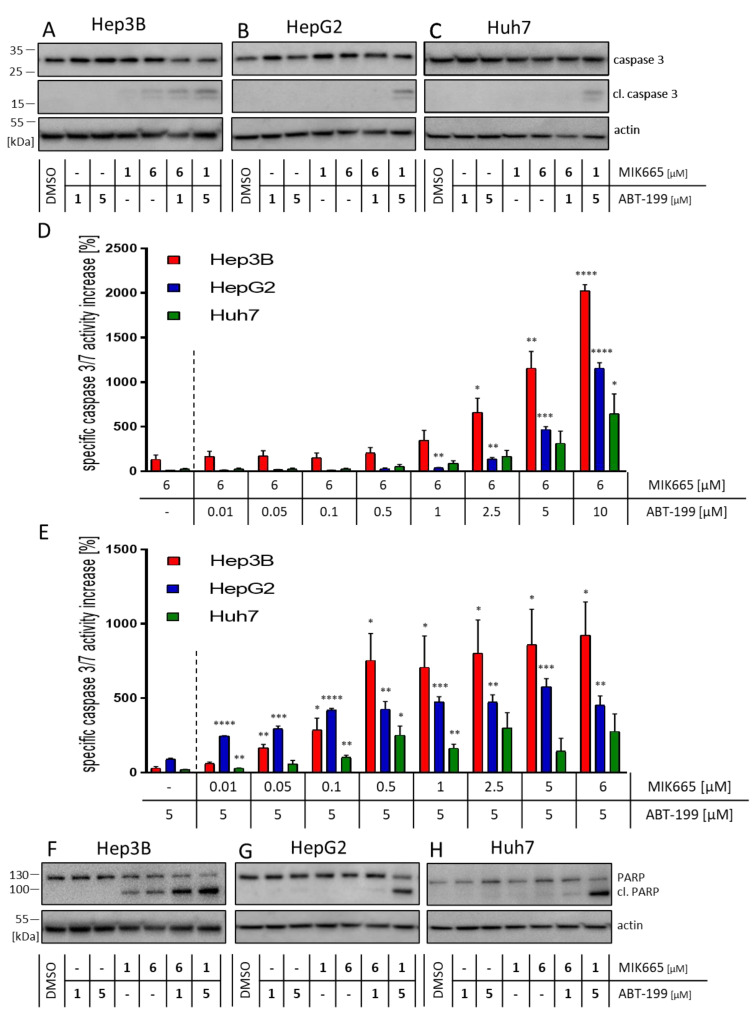
Combination of ABT-199 and MIK665 results in increased cleavage and activity of caspase-3 in HCC cell lines. (**A**–**C**) Western blot analyses of (cleaved) caspase-3. Shown is a representative western blot. Cells were treated with either one of the two BH3-mimetics ABT-199 or MIK665 alone or a combination of both inhibitors. DMSO was used as vehicle control. (**D**,**E**) Activity assays of caspase-3/7 in HCC cell lines (Hep3B (red), HepG2 (blue), and Huh7 (green)) after 4 h of treatment with either ABT-199 or MIK665 alone or in combination at the indicated concentrations (the dotted line separates the single treatment from the combination treatment with ABT-199 and MIK665). Data show a specific increase in activity compared to DMSO-treated controls (*n* = 3, mean ± SEM). (**F**–**H**) Western blot analyses of (cleaved) PARP. Shown is a representative western blot. Cells were incubated with ABT-199 and MIK665 at the indicated concentrations as single or combined treatment. DMSO was used as vehicle control. (* *p* < 0.05; ** *p* < 0.01; *** *p* < 0.001; **** *p* < 0.0001 compared to single drug treatment).

Active caspase-9 activates the effector caspase-3 by proteolytic cleavage. As assumed, treatment with ABT-199 and MIK665 alone resulted in a rather low induction of cleavage and activity of caspase-3 (Figure 4A–E). Of note, the combination of ABT-199 and MIK665 led to increased caspase-3 cleavage in all cell lines (Figure 4A–E). After 24 h of treatment, caspase-3 activity was still enhanced in samples treated with the combination of the inhibitors compared to single drug-treated cells (Supplementary Figure S4A–F).

To further investigate the potential involvement of the extrinsic apoptosis pathway upon inhibition of Bcl-2 and Mcl-1, we analyzed the activity of caspase-8. No relevant involvement of caspase-8, and thus no extrinsic apoptosis, was detected upon treatment with the BH3-mimetics (Supplementary Figure S5A–F). ABT-199 and MIK665 induce the intrinsic apoptosis pathway. Activation of effector caspase-3 leads to the cleavage of so-called death substrates, such as poly(ADP-ribose)polymerase-1 (PARP) [49]. After combined treatment with both inhibitors, PARP cleavage was observed in all cell lines examined. Single-drug treatment only resulted in discreet PARP cleavage in MIK665-treated Hep3B cells (Figure 4F–H). Taken together, our experiments show an induction of the intrinsic apoptosis pathway in HCC cell lines after combination treatment with ABT-199 and MIK665. The combined inhibition of two anti-apoptotic BCL-2 family members, namely Bcl-2 and Mcl-1, was demonstrated to effectively induce the caspase cascade.

### 3.4. The Combination of ABT-199 and MIK665 Effectively Induces Cell Death in HCC Cell Lines

**Figure 5:**
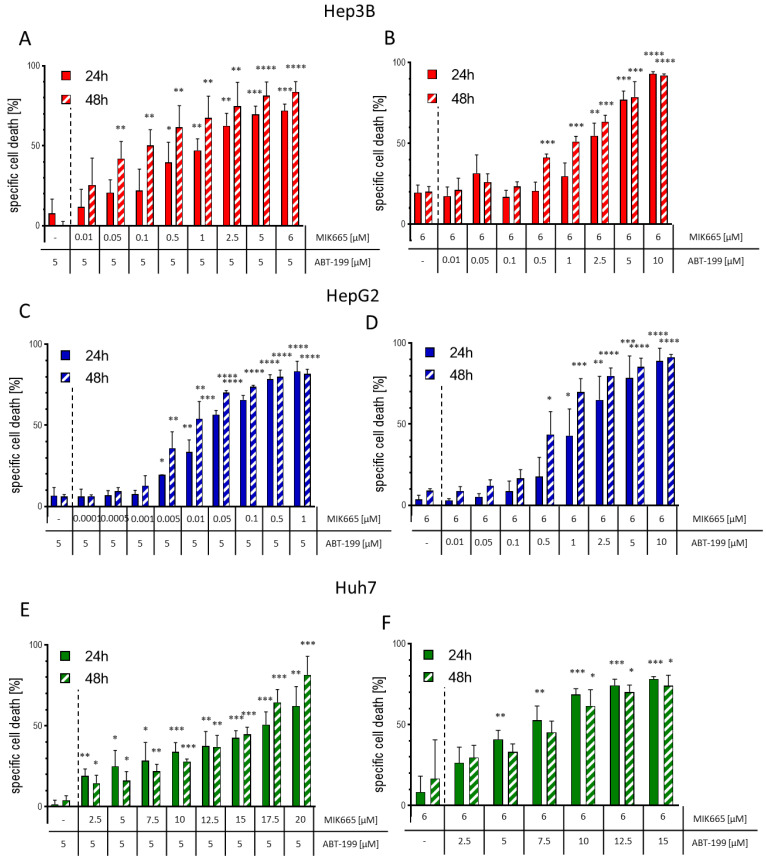
Treatment with combinations of ABT-199 and MIK665 induces cell death in Hep3B, HepG2 and Huh7. (**A**,**C**,**E**) Specific cell death was analyzed in HCC cells treated with a constant ABT-199 concentration (5 µM) and increasing MIK665 concentrations as indicated for up to 48 h (*n* = 3, mean ± SD). (**B**,**D**,**F**) Specific cell death was analyzed in HCC cells treated with a steady MIK665 concentration (6 µM) and increasing ABT-199 concentrations as indicated for up to 48 h (the dotted line separates the single treatment from the combination treatment with ABT-199 and MIK665) (*n* = 3, mean ± SD) (* *p* < 0.05; ** *p* < 0.01; *** *p* < 0.001; **** *p* < 0.0001 compared to single drug treatment).

Since the combination treatment of ABT-199 and MIK665 induces the caspase cascade, it can be assumed that this combination can successfully induce apoptosis in HCC cells. Therefore, we evaluated the impact of combination treatment on specific cell death using flow cytometry. Cells were treated with a constant concentration of ABT-199 (5 µM) and varying MIK665 concentrations, or vice versa, with 6 µM MIK665 and varying concentrations of ABT-199. As shown in our previous results, single treatment with ABT-199 and MIK665 induced low cell death rates. The combination of both drugs resulted in significantly increased cell death in a dose-dependent manner (Figure 5A–F, Supplementary Figures S6A–E, S7A–F and S8A–E, Supplementary Table S1). Hence, cell death induction could be increased up to fivefold by combination treatment compared to a single treatment. The extent of cell death induction was dependent on the cell line. Strongest effects were measured in HepG2. Notably, a concentration of 5 µM ABT-199 and only low nanomolar concentrations of MIK665 were sufficient to induce high cell death rates in HepG2 cells (Figure 5C,D). Huh7 cells, on the other hand, displayed the lowest effects after treatment (Figure 5E,F). Induction of cell death does not correlate with the expression of other BCL-2 family members, such as Bcl-X_L_ and NOXA. High Bcl-X_L_ expression in HepG2 cells could not block apoptosis, and the expression of NOXA seen in HepG2 and Huh7 cells did not result in enhancement of cell death induction by MIK665 and ABT-199 (Figure 1D,E,H,I). In addition, we examined whether cell death induced by the combination of ABT-199 and MIK665 could be blocked by the pan-caspase inhibitor zVAD. Co-treatment with ABT-199/MIK665 combinations and zVAD reduced cell death induction after 24 h in all analyzed HCC cell lines compared to treatment without zVAD (Supplementary Figure S9A–F). Our data suggest that the combination of ABT-199 and MIK665 effectively induces caspase-dependent intrinsic apoptosis in HCC cells.

### 3.5. ABT-199 and MIK665 Act Synergistically in HCC Cell Lines

**Figure 6:**
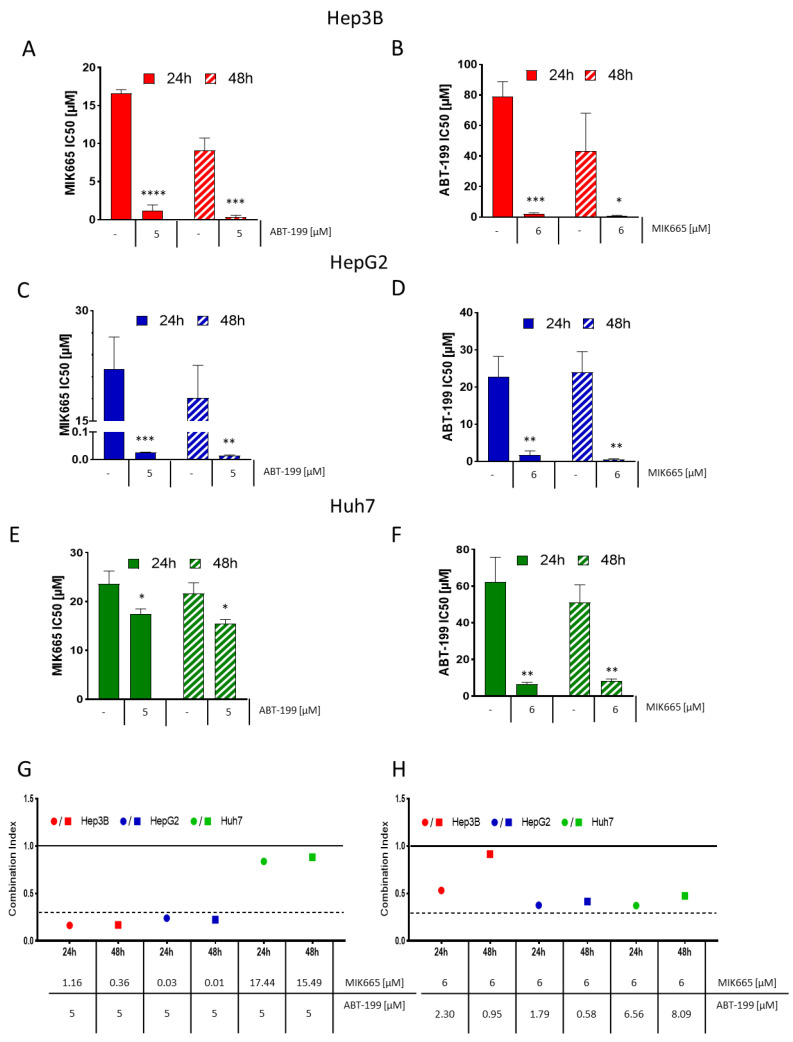
Combination treatment with ABT-199 and MIK665 has synergistic effects in HCC cells. (**A**–**F**) Graphs show the IC_50_ of monotherapy and combinations of ABT-199 and MIK665 administered to the HCC cell lines Hep3B, HepG2 and Huh7 for up to 48 h of treatment. For combination treatments, all cell lines were treated with a steady concentration of 5 µM ABT-199 or 6 µM MIK665 and increasing concentrations of the respective other BH3-mimetic (*n* = 3, mean ± SD). IC_50_ calculation was executed with GraphPad Prism 8 (* *p* <0.05; ** *p* <0.01; *** *p* <0.001; **** *p* < 0.0001 compared to single drug treatment). (**G**,**H**) Graphs show dot plots of the calculated combination indices (CI) of the interpolated IC_50_ of the ABT-199/MIK665 combinations at 24 h and 48 h of treatment. CI was calculated using CompuSyn (version 1) (http://www.combosyn.com/ (accessed on 26 May 2023)). CI values < 1 indicate a synergistic effect of the combination treatment. Smaller CI values indicate stronger synergism. A CI < 0.3 (dotted line) signifies strong to very strong synergism [44].

Due to the impressive effect of the combination of BH3-mimetics, we further investigated whether both drugs work in an additive or synergistic manner. Therefore, we determined the half-maximum inhibitory concentration (IC_50_) for ABT-199 and MIK665 alone and in combination for each HCC cell line after 24 h and 48 h of treatment. The IC_50_ of the combination treatments was assessed using a constant concentration of 5 µM ABT-199 and increasing concentrations of MIK665, or vice versa, with 6 µM MIK665 and increasing concentrations of ABT-199. The IC_50_ of both ABT-199 and MIK665 alone was comparatively high (18.2 µM–23.6 µM after 24 h treatment and 10.1 µM–21.6 µM after 48 h, Figure 6A–F, Supplementary Figures S10A,B, S11A,B and S12A,B). At a constant ABT-199 concentration of 5 µM, the IC_50_ of MIK665 decreased to 0.03 µM–17.4 µM after 24 h and to 0.01 µM–15.5 µM after 48 h of treatment.

At a constant concentration of 6 µM MIK665, the IC_50_ of ABT-199 decreased to 1.8–6.6 µM after 24 h and to 0.6–8.1 µM after 48 h of treatment. Using the CompuSyn software, we calculated the Combination Index (CI) according to Chou et al. [44]. This dimensionless value describes the synergistic (CI < 1), additive (CI = 1), or antagonistic (CI > 1) effect of a combination of substances versus either substance alone. Our analyses demonstrate synergistic effects of ABT-199 and MIK665 combinations in all analyzed cell lines (Figure 6G,H, Supplementary Figures S10C,D, S11C,D and S12C,D). A CI < 0.3 (dotted line) demonstrates strong synergism of the combination of MIK665 and ABT-199 in Hep3B and HepG2 at the indicated concentrations after 24 h and 48 h of treatment. A CI < 1 (solid line) demonstrates synergism of the combination of MIK665 and ABT-199 in all analyzed cell lines after 24 h and 48 h. It can be concluded that inhibition of both Bcl-2 and Mcl-1 is necessary to induce apoptosis in HCC cells efficiently. Only combined blockage of these two proteins effectively induces cytochrome c release, induction of the caspase cascade, and, finally, apoptosis.

## 4. Discussion

Although the major risk factors for HCC pathogenesis are well known, the incidence and mortality rates of HCC remain high, with the incidence steadily rising [26,44,45]. In North America and Europe, more than 60% of HCC are first diagnosed in intermediate (BCLC-B) or advanced stages (BCLC-C and -D) [50]. Despite recent developments leading to new treatment options, the prognosis for advanced-staged HCC is still poor [21,51]. The combination of atezolizumab and bevacizumab as first-line therapy for HCC improved median overall survival (OS) (to 19.2 months) and progression-free survival (6.8 months, compared to 4.3 months with sorafenib) for HCC patients [23,52]. Recently, the immune checkpoint inhibitors tremelimumab (anti-CTLA-4) and durvalumab (anti-PD-L1) were approved as another first-line combination regimen by the FDA and EMA. This regimen improved median OS compared to sorafenib, as shown in the HIMALAYA trial. Median OS was 16.4 months with the combination, compared to 13.8 months with sorafenib. In the LEAP-002 study (NCT03713593), the combination of lenvatinib with pembrolizumab showed promising anti-tumor activity with a median OS of 21.2 months. However, it failed to be superior to lenvatinib (median OS: 19.0 months) [53]. Despite this progress, existing therapies still have low overall response rates, and most patients have progress of the disease. Thus, new treatment options after the failure of existing therapies are urgently needed [54].

BH3-mimetics are promising therapeutic options for systemic anti-tumor treatment [15,30]. Several Mcl-1 inhibitors are currently being investigated in clinical trials for various hematological malignancies. For example, MIK665 is under investigation for the treatment of relapsed and/or refractory patients with lymphoma or multiple myeloma (NCT02992483 clinical study phase I) [55]. Another clinical trial analyzes the potential of MIK665 in patients with acute myeloid leukemia or myelodysplastic syndrome (NCT04629443 clinical study phase I/II) [56]. AMG176, another Mcl-1 inhibitor, is being tested in participants with relapsed or refractory multiple myeloma, and in patients with relapsed or refractory acute myeloid leukemia (NCT02675452 clinical study phase I) [57]. The Mcl-1 inhibitor PRT1419 is being investigated for the treatment of patients with relapsed and/or refractory hematologic malignancies (NCT04543305, clinical phase I) [58].

The BH3-mimetic ABT-199/venetoclax is already approved and in clinical use in the therapy of AML [10]. Initial studies with ABT-199 in CLL showed promising results. A phase I trial (NCT01328626) in patients with relapsed or refractory CLL reported overall response rates of 79% and complete response rates of 20%. Further studies led to the approval of ABT-199 for CLL treatment in 2016 [10,11,59]. ABT-199-based therapy has been reported to be tolerated much better and to be more effective than conventional chemo-immunotherapy in patients with CLL or AML, thus providing patients with a higher quality of life [60]. Resistance to therapy is still a major issue.

One mechanism of resistance to Bcl-2 inhibition is the upregulation of Mcl-1 [29,48]. An ongoing clinical trial currently evaluates the therapeutic effects of the combination of ABT-199 and MIK665 (NCT03672695) in AML [61]. The same combination also showed promising results in T cell lymphoblastic leukemia cells [62]. Most solid tumors, including HCC, depend on the expression of more than one BCL-2 molecule, supporting the idea to combine different BH3-mimetics [63]. Solid tumors have been shown to respond to ABT-199/MIK665 combinations [30]. For example, Mukherjee et al. showed that the combination of ABT-199 and MIK665 led to reduced cell viability and an increased PARP cleavage in different melanoma cell lines. ABT-199/MIK665 treatment was also effective in a corresponding mouse model, leading to tumor volume reduction with minimal toxicity. Seiller et al. showed efficacy of the same combination in multiple myeloma [43,64]. Various malignancies, including HCC, express Mcl-1 at a significantly higher level compared to healthy cells [65]. Mcl-1 gene amplifications are often found in human cancer. Mcl-1 appears to be a key factor for the resistance to conventional cancer therapies in some cancer types [66]. Targeting Mcl-1 simultaneously with Bcl-2 restores sensitivity toward ABT-199 in AML cells [67]. Therefore, we hypothesize that a combination of the selective Mcl-1 inhibitor MIK665 with ABT-199 could also be very effective in HCC.

Here, we present a possible new treatment option for HCC. Our data provide evidence that simultaneously targeting Bcl-2 and Mcl-1 with BH3-mimetics is successful across different HCC cell lines, irrespective of the p53 status (HepG2: wtp53, Hep3B: deleted p53 and Huh7: mutated p53) and Bcl-X_L_ and NOXA expression. This was characterized by the release of cytochrome c, cleavage and activation of initiator caspase-9 and executioner caspase-3, and the cleavage of the apoptosis marker PARP. Both inhibitors act synergistically, and thus offer the possibility of bypassing existing protective mechanisms of the tumor. The inhibitors show little effect individually, but the combination effectively induces apoptosis in HCC cells. This indicates that cellular survival co-depends on Bcl-2 and Mcl-1. Cells that respond to simultaneous inhibition of multiple BCL-2 molecules do not necessarily overexpress all of these proteins [15,68]. Therefore, we cannot directly conclude the treatment response from the expression pattern of BCL-2 molecules in our cells. However, Hep3B and HepG2 cells, which demonstrated the highest rates of increase in caspase activity and cell death, had high amounts of at least one of Bcl-2 or Mcl-1 compared to the other cell lines. In contrast, Huh7 cells expressed low amounts of both proteins. Therefore, Huh7 cells are likely less dependent on these survival pathways, which may explain why these cells were less sensitive to the treatment used than the other HCC cell lines. Nevertheless, all cell lines showed synergistic effects upon drug combination, indicating co-dependencies of the cell lines on Bcl-2 and Mcl-1. This is in agreement with synergistic effects also demonstrated in different melanoma cell lines when ABT-199 and MIK665 were combined [43].

HCC cells overexpress anti-apoptotic proteins such as Bcl-2, or Mcl-1 in a heterogeneous manner [69]. Therefore, expression profiling of these resistance factors in HCC patients can help to develop new treatment options. Selecting patients with specific expression patterns or testing responsiveness before treatment using BH3 profiling could significantly increase treatment success [70]. Furthermore, BH3-mimetics might be used to enhance the efficacy of transarterial chemoembolization (TACE) in the treatment of intermediate-stage HCC. The combination with this local treatment could potentially reduce the inhibitor concentration needed.

BH3-mimetics bypass mutations in upstream signaling pathways. Both ABT-199 and MIK665 induce apoptosis in cells without an intact p53 tumor-suppressor pathway [18,71]. This is in line with our results showing that the application works in Hep3B cells, which do not express p53, as well as in HepG2, which express a wild type of p53 (Figure 1A). Using a combination of ABT-199 and MIK665 could therefore be a viable approach for HCC patients displaying a p53 mutation. Mutations of p53 occur in 25–30% of patients with HCC, and lead to shorter median OS and relapse-free survival times compared to patients with wild-type p53 [72,73]. The use of BH3-mimetics bypasses p53 mutations to successfully induce apoptosis.

In summary, our data indicate that the combination of two different BH3-mimetics induces apoptosis synergistically in HCC cell lines. This opens up new strategies for systemic treatment of HCC. Resistance can potentially be circumvented, and the dose of drugs can be reduced by combining them. This also reduces the risk of serious side effects. Therefore, the combination of BH3-mimetics presented here may represent an important step towards the future development and evaluation of a new treatment option in HCC.

## Data Availability

Not applicable.

## Supplementary Materials

The following supporting information can be downloaded at: https://www.mdpi.com/article/10.3390/biomedicines11061666/s1, Supplementary Figure S1. Combination of ABT-199 and MIK665 leads to decreased mitochondrial membrane potential in Hep3B. Figure S2: Combination of ABT-199 and MIK665 results in increased cytochrome c release in Hep3B cells. Supplementary Figure S3: Caspase-9 activity is elevated up to 6 h after treatment with an ABT-199 and MIK665 combination. Supplementary Figure S4: Caspase-3/7 activity remains significantly enhanced after 24 h of treatment with a BH3-mimetics combination. Supplementary Figure S5: Caspase 8 (extrinsic apoptosis signaling pathway) is not or only to a very low extent induced by application of BH3-mimetics. Supplementary Figure S6: The combination of ABT-199 and MIK 665 increases cell death in Hep3B after 24 h treatment. Supplementary Figure S7: The combination of ABT-199 and MIK665 increases cell death rates in HepG2 after 24 h. Supplementary Figure S8: The combination of ABT-199 and MIK665 increases cell death in Huh7 after 24 h treatment. Supplementary Table S1: Summary of flow cytometry analysis shown in Figures S6–8. Supplementary Figure S9: The pan-caspase inhibitor zVAD inhibits cell death induction by ABT-199/MIK665. Supplementary Figure S10: Combination of BH3-mimetics leads to induction of synergistic cell death in Hep3B. Supplementary Figure S11: Combination of BH3-mimetics leads to induction of synergistic cell death in HepG2. Supplementary Figure S12: Combination of BH3-mimetics leads to induction of synergistic cell death in Huh7. Supplementary Methods - Determination of mitochondrial membrane potential.
