# Supplementary material for: Simultaneous Inhibition of Mcl-1 and Bcl-2 Induces Synergistic Cell Death in Hepatocellular Carcinoma
Source: Biomedicines. 2023 Jun 8;11(6):1666. doi: 10.3390/biomedicines11061666 (PMC10295989; doi:10.3390/biomedicines11061666)
Supplement: Supplementary file 1 [file biomedicines-11-01666-s001.zip › biomedicines-2398916-supplementary.pdf]

Supplementary Data

Simultaneous Inhibition of Mcl-1 and Bcl-2 Induces Synergistic Cell Death in Hepatocellular Carcinoma

Table of Content

Content	Page No.
Figure S1. Combination of ABT-199 and MIK665 leads to decreased mitochondrial membrane potential in Hep3B.	p. 2
Figure S2. Combination of ABT-199 and MIK665 results in increased cytochrome c release in Hep3B cells.	p. 3
Figure S3. Caspase 9 activity is increased 6 h after treatment with an ABT 199 and MIK665 combination.	p. 4
Figure S4 Caspase 3/7 activity remains significantly enhanced after 24 h of treatment with a BH3-mimetics combination.	p. 5
Figure S5. Caspase 8 (extrinsic apoptosis signaling pathway) is not or only to a very low extent induced by application of BH3-mimetics.	p. 6
Figure S6. The combination of ABT-199 and MIK665 increases cell death in Hep3B after 24 h treatment.	p. 7
Figure S7. The combination of ABT-199 and MIK665 increases cell death in HepG2 after 24 h treatment.	p. 8
Figure S8. The combination of ABT-199 and MIK665 increases cell death in Huh7 after 24 h treatment.	p. 9
Table S1: Summary of flow cytometry analysis shown in Figures S6-8.	p. 10
Figure S9. The pan-caspase inhibitor zVAD inhibits cell death induction by ABT-199/MIK665.	p. 11
Figure S10. Combination of BH3-mimetics leads to induction of synergistic cell death in Hep3B.	p. 12
Figure S11. Combination of BH3-mimetics leads to induction of synergistic cell death in HepG2.	p. 13
Figure S12. Combination of BH3-mimetics leads to induction of synergistic cell death in Huh7.	p. 14
Supplementary Methods - Determination of mitochondrial membrane potential	p 15

Supplementary Figures

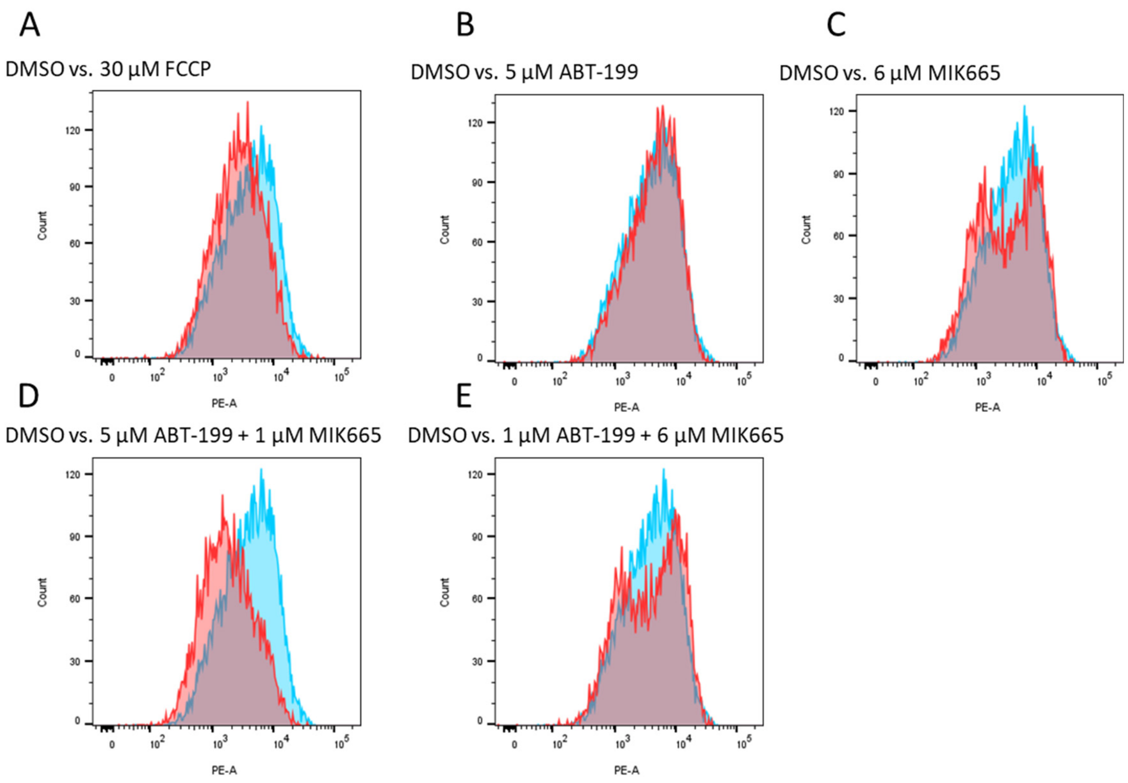

Figure S1. Combination of ABT-199 and MIK665 leads to decreased mitochondrial membrane potential in Hep3B. TMRE-staining of Hep3B after 8 h of treatment with BH3-mimetics. DMSO (blue), treatment (red). (**A**) Cells were treated with 30 µM FCCP for 3 h to induce MOMP as positive control. (**B-E**) TMRE-staining of Hep3B cells. Cells were subjected to ABT-199 and MIK665 for 8 h at the indicated concentrations as single or combined treatment (n=3).

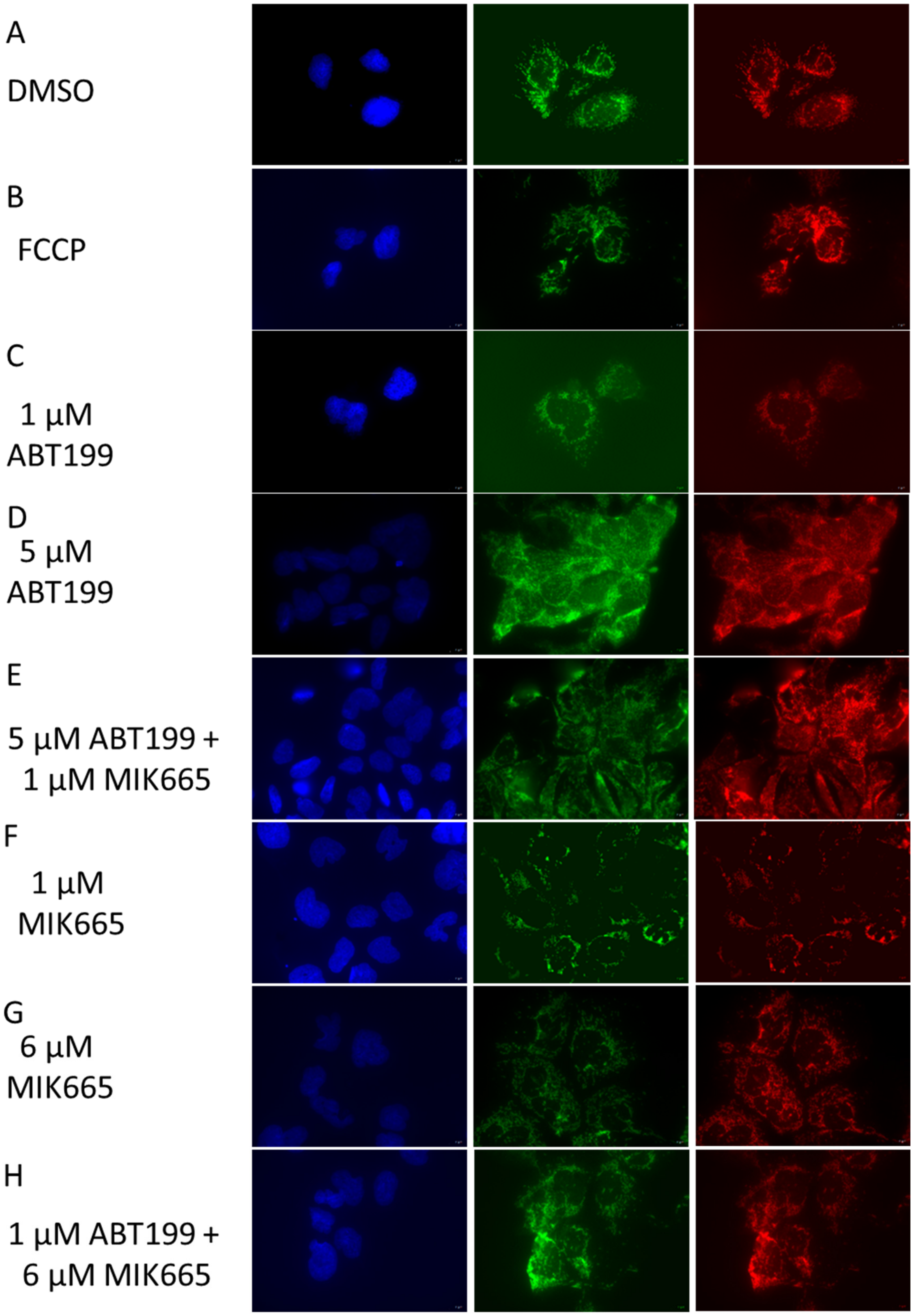

Figure S2. Combination of ABT-199 and MIK665 results in increased cytochrome c release in Hep3B cells. Immunofluorescence of DAPI (blue), TOMM20 (green) and cytochrome c (red). (**A**) DMSO treatment was used as vehicle control. (**B**) Cells were treated with 30 µM FCCP for 3 h to induce cytochrome c release as positive control. (**C-I**) Detection of TOMM20 (mitochondrial marker) and cytochrome c in Hep3B cells. Cells were subjected to ABT-199 and MIK665 for 4 h at the indicated concentrations as single or combined treatment (n=3).

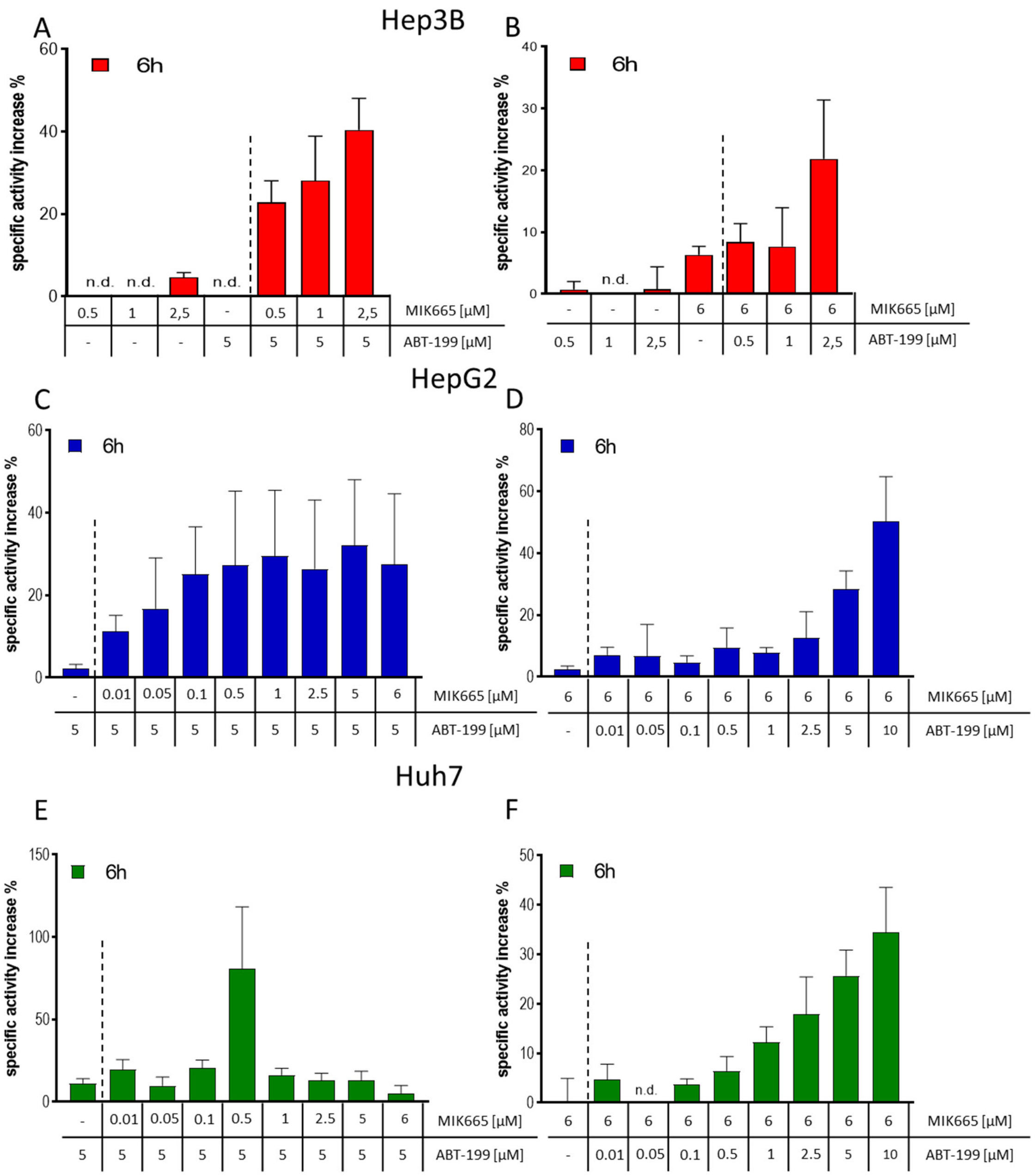

Figure S3. Caspase 9 activity is increased 6 h after treatment with an ABT-199 and MIK665 combination. (**A**, **C**, **E**) Activity assays of caspase 9 in HCC cell lines after 6 h of treatment with 5 µM ABT-199 and increasing MIK665 concentrations. Data show a specific increase in activity compared to DMSO-treated controls (n=3, mean±SEM). (**B**, **D**, **F**) Activity assays of caspase 9 in HCC cell lines after 6 h of treatment with 6 µM MIK665 and increasing ABT-199 concentrations. Data show a specific increase in activity compared to DMSO-treated controls (n=3, mean±SEM) (the dotted line separates the single treatment from the combination treatment with ABT-199 and MIK665).

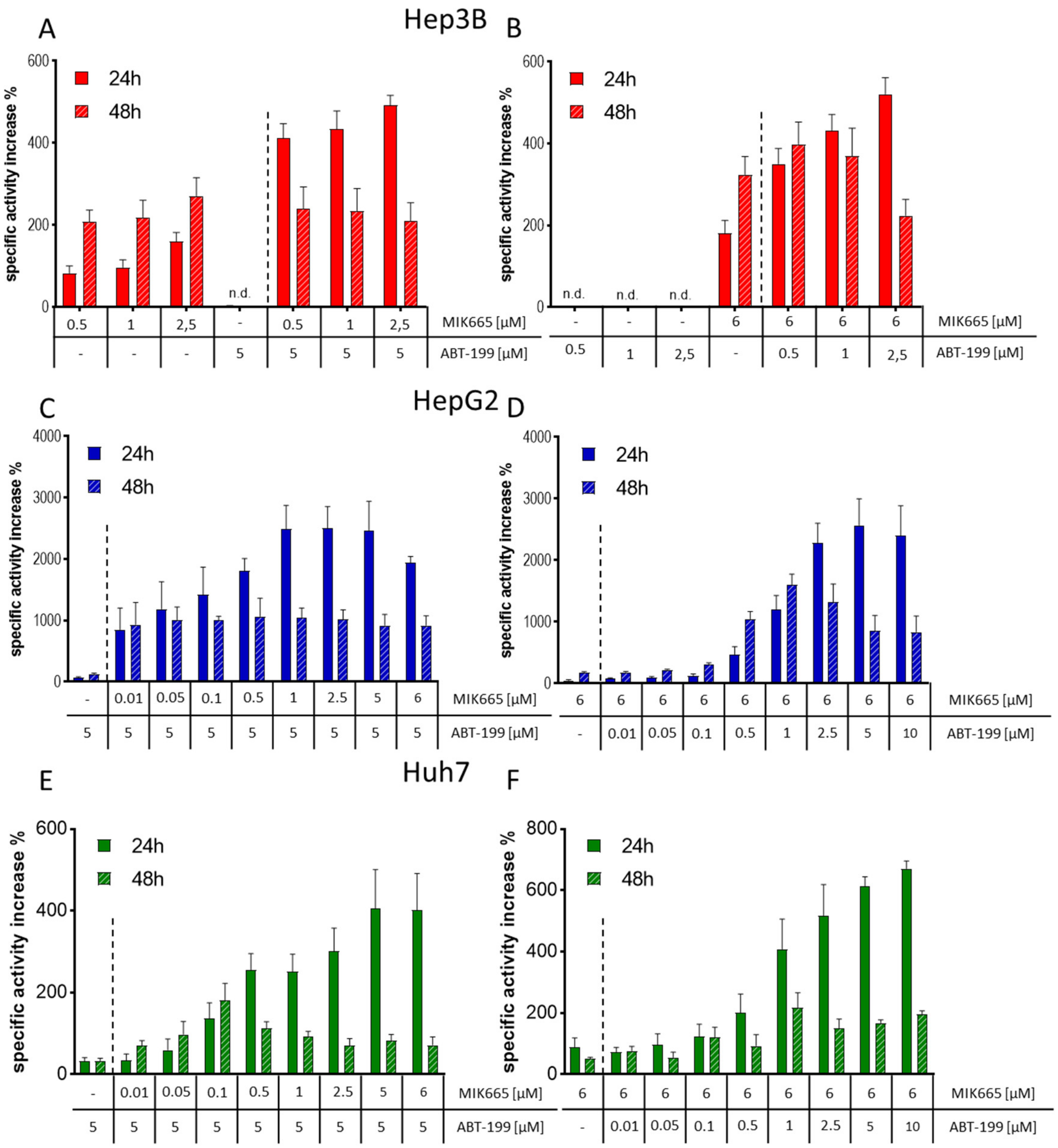

Figure S4. Caspase 3/7 activity remains significantly enhanced after 24 h of treatment with a BH3-mimetics combination. (**A**, **C**, **E**) Activity assays of caspase 3/7 in HCC cell lines after 24 h and 48 h of treatment with 5 µM ABT-199 and increasing MIK665 concentrations. Data show a specific increase in activity compared to DMSO-treated controls (n=3, mean±SEM). (**B**, **D**, **F**) Activity assays of caspase 3/7 in HCC cell lines after 24 h and 48 h of treatment with 6 µM MIK665 and increasing ABT-199 concentrations. Data show a specific increase in activity compared to DMSO-treated controls (n=3, mean±SEM) (the dotted line separates the single treatment from the combination treatment with ABT-199 and MIK665).

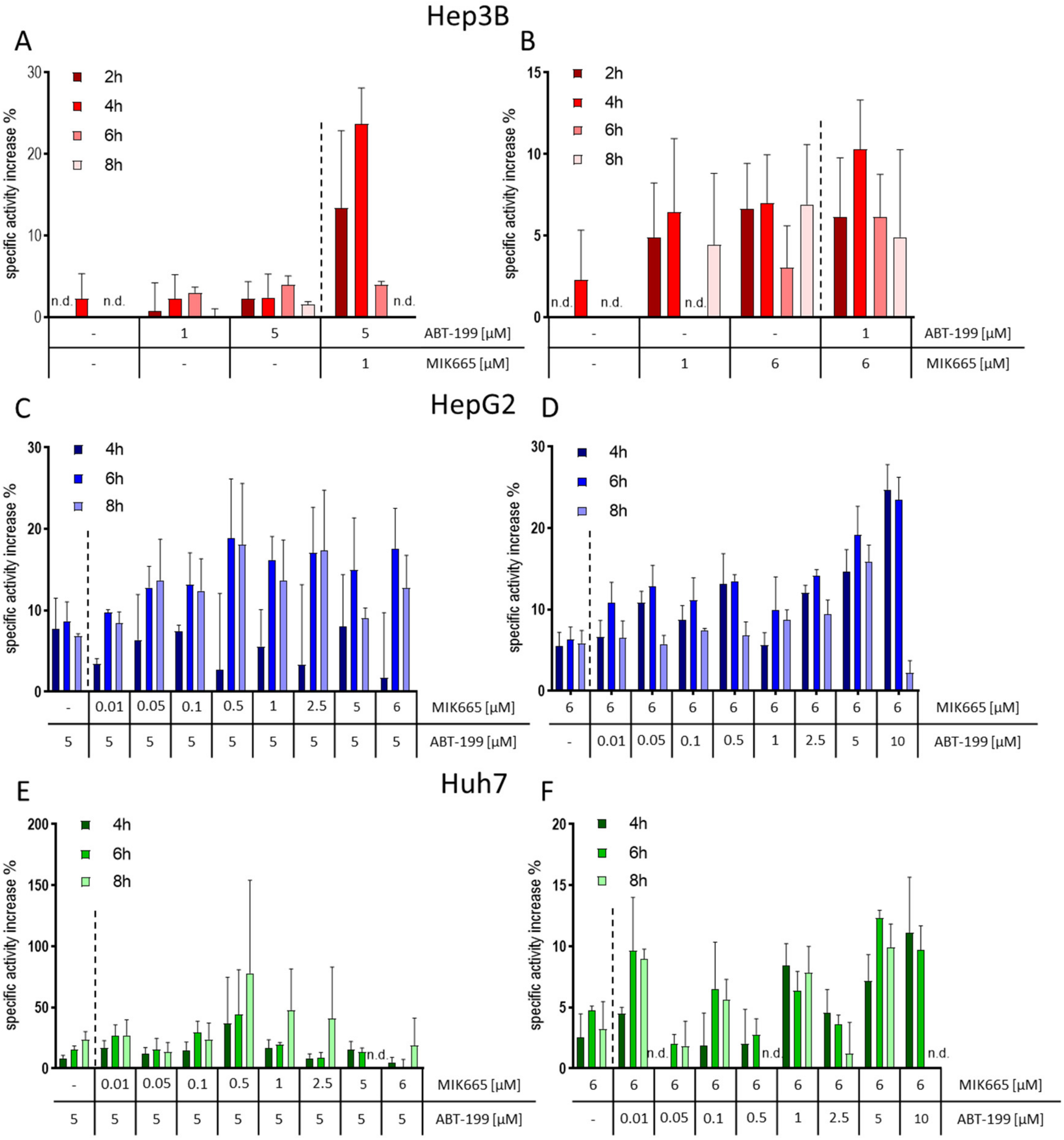

Figure S5. Caspase 8 (extrinsic apoptosis signaling pathway) is not or only to a very low extent induced by application of BH3-mimetics. (**A**, **C**, **E**) Activity assays of caspase 8 in HCC cell lines after 4 h – 8 h of treatment with 5 µM ABT-199 and increasing MIK665 concentrations. Data show a specific increase in activity compared to DMSO-treated controls (n=3, mean±SEM). (**B**, **D**, **F**) Activity assays of caspase 8 in HCC cell lines after 4 h – 8 h of treatment with 6 µM MIK665 and increasing ABT-199 concentrations. Data show a specific increase in activity compared to DMSO-treated controls (n=3, mean±SEM) (the dotted line separates the single treatment from the combination treatment with ABT-199 and MIK665).

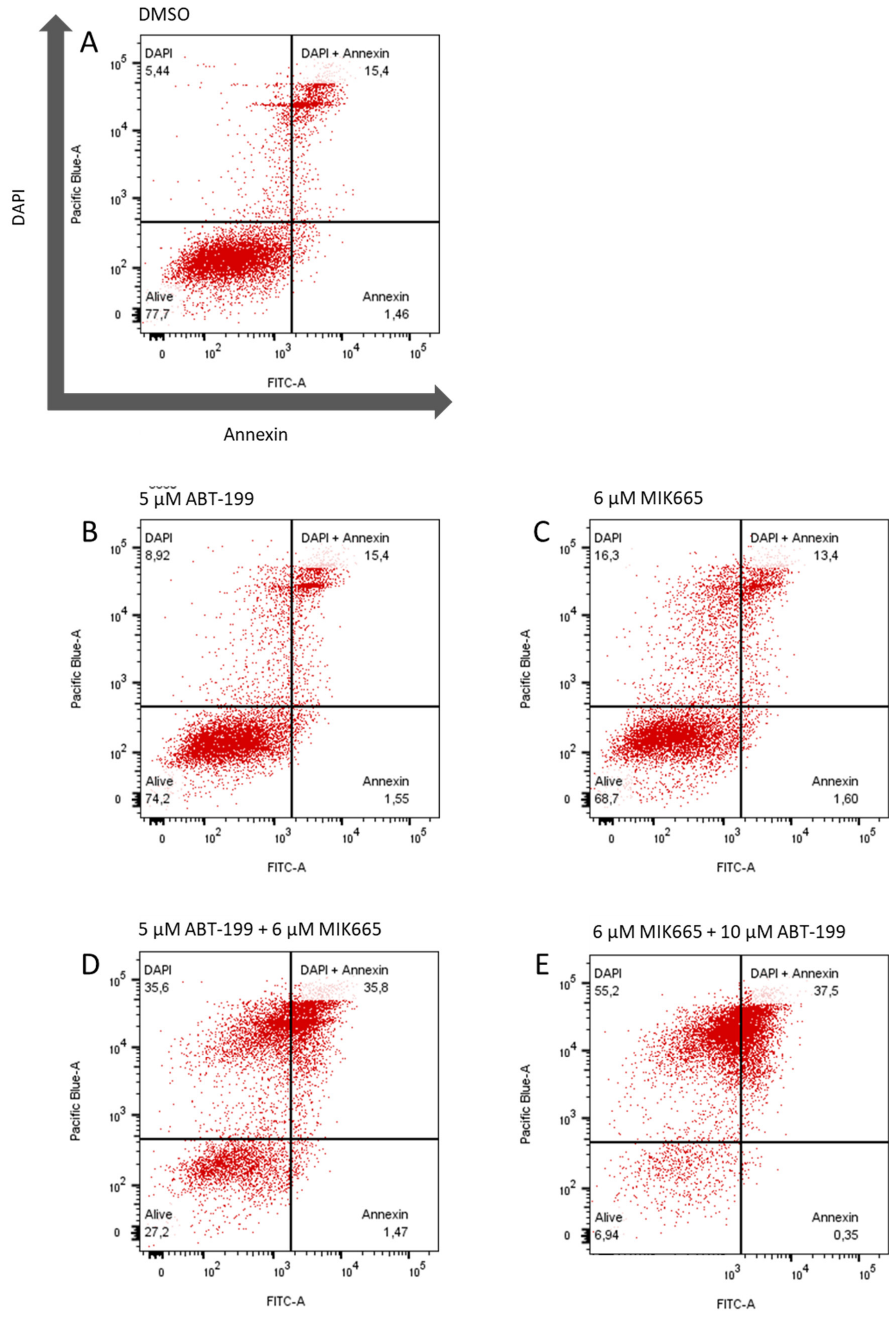

Figure S6. The combination of ABT-199 and MIK665 increases cell death in Hep3B after 24 h treatment. Hep3B cells were stimulated as indicated and stained with Annexin V-FiTC and DAPI, and analyzed by flow cytometry. (**A**) DMSO treated control. (**B**) Treatment with 5 µM ABT-199. (**C**) Treatment with 6 µM MIK665. (**D**) Treatment with 5 µM ABT-199 and 6 µM MIK665. (**E**) Treatment with 6 µM MIK665 and 10 µM MIK665.

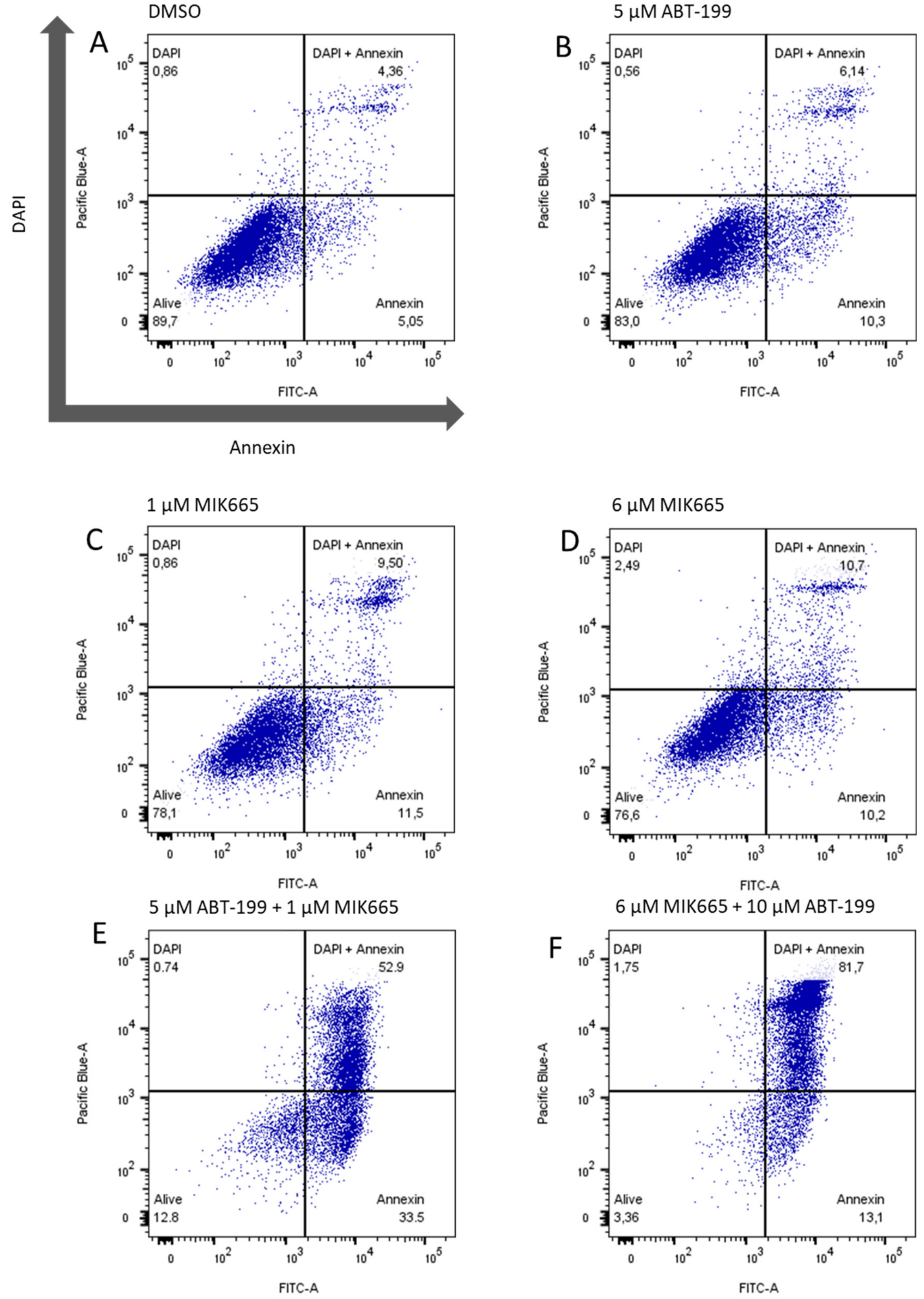

Figure S7. The combination of ABT-199 and MIK665 increases cell death in HepG2 after 24 h treatment. HepG2 cells were stimulated as indicated and stained with Annexin V-FiTC and DAPI, and analyzed by flow cytometry. (**A**) DMSO treated control. (**B**) Treatment with 5 µM ABT-199. (**C**) Treatment with 1 µM MIK665. (**D**) Treatment with 6 µM MIK665. (**E**) Treatment with 5 µM ABT-199 and 1 µM MIK665. (**F**) Treatment with 6 µM MIK665 and 10 µM MIK665.

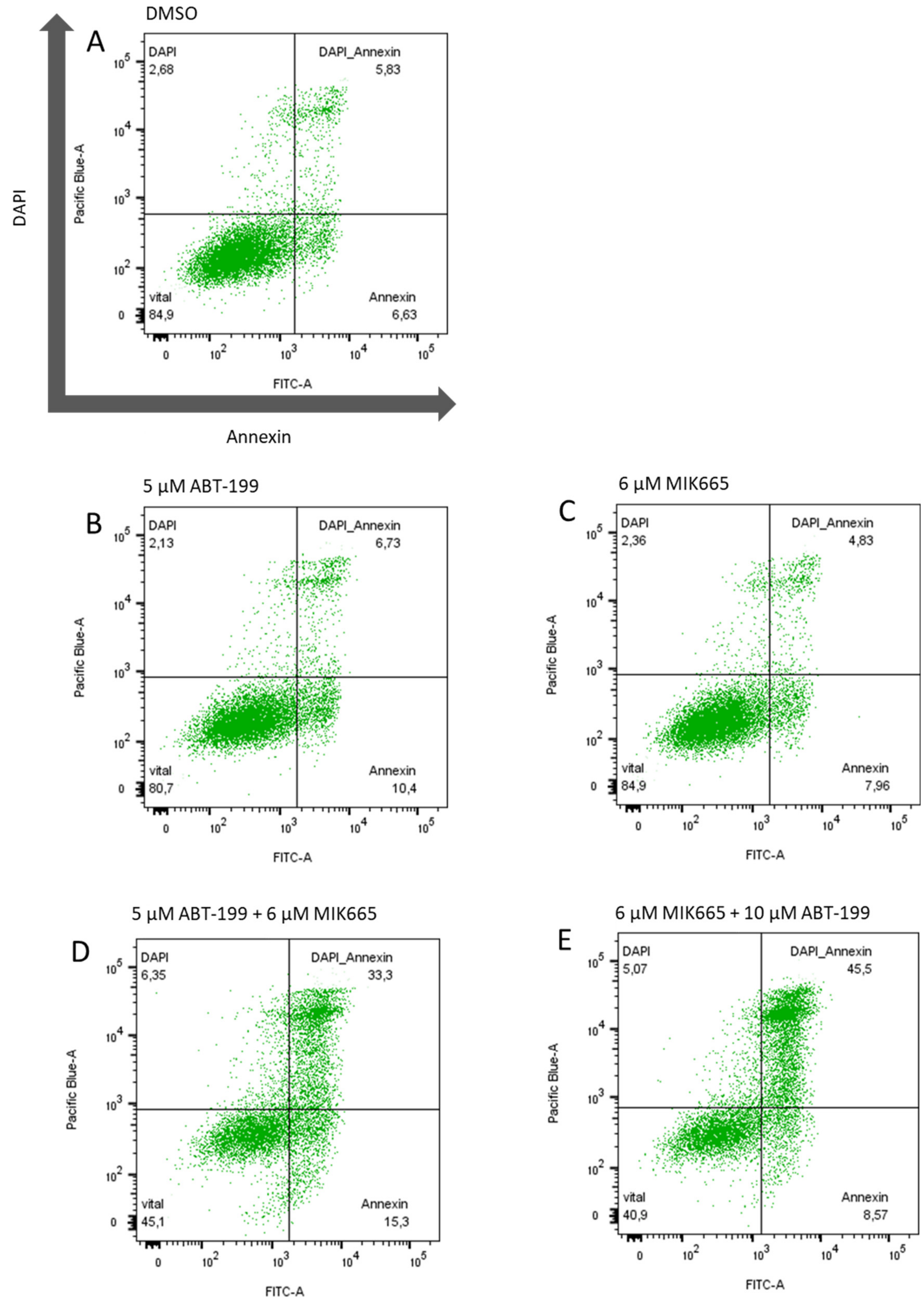

Figure S8. The combination of ABT-199 and MIK665 increases cell death in Huh7 after 24 h treatment. Huh7 cells were stimulated as indicated and stained with Annexin V-FiTC and DAPI, and analyzed by flow cytometry. (**A**) DMSO treated control. (**B**) Treatment with 5 μM ABT-199. (**C**) Treatment with 6 μM MIK665. (**D**) Treatment with 5 μM ABT-199 and 6 μM MIK665. (E) Treatment with 6 μM MIK665 and 10 μM MIK665.

Table S1: Summary of flow cytometry analysis shown in Figures S6-8. Cells that were not positive for either Annexin V staining or DAPI (nuclear staining) were considered alive. Cells that were positive for Annexin V staining or DAPI staining and cells that were positive for both stainings were considered dead.

Cell line	Treatment	vital [%]	Dead [%]
**Hep3B**	DMSO	77.7	22.3
	5 µM ABT-199	74.2	25.87
	6 µM MIK665	68.7	31.3
	5 µM ABT-199 + 6 µM MIK665	27.2	72.87
	6 µM MIK665 + 10 µM ABT-199	6.94	93.05
**HepG2**	DMSO	89.7	10.27
	5 µM ABT-199	83	17
	1 µM MIK665	78.1	21.86
	6 µM MIK665	76.6	23.39
	5 µM ABT-199 + 1 µM MIK665	12.8	87.14
	6 µM MIK665 + 10 µM ABT-199	3.36	96.55
**Huh7**	DMSO	84.9	15.14
	5 µM ABT-199	80.7	19.26
	6 µM MIK665	84.9	15.15
	5 µM ABT-199 + 6 µM MIK665	45.1	54.95
	6 µM MIK665 + 10 µM ABT-199	40.9	59.14

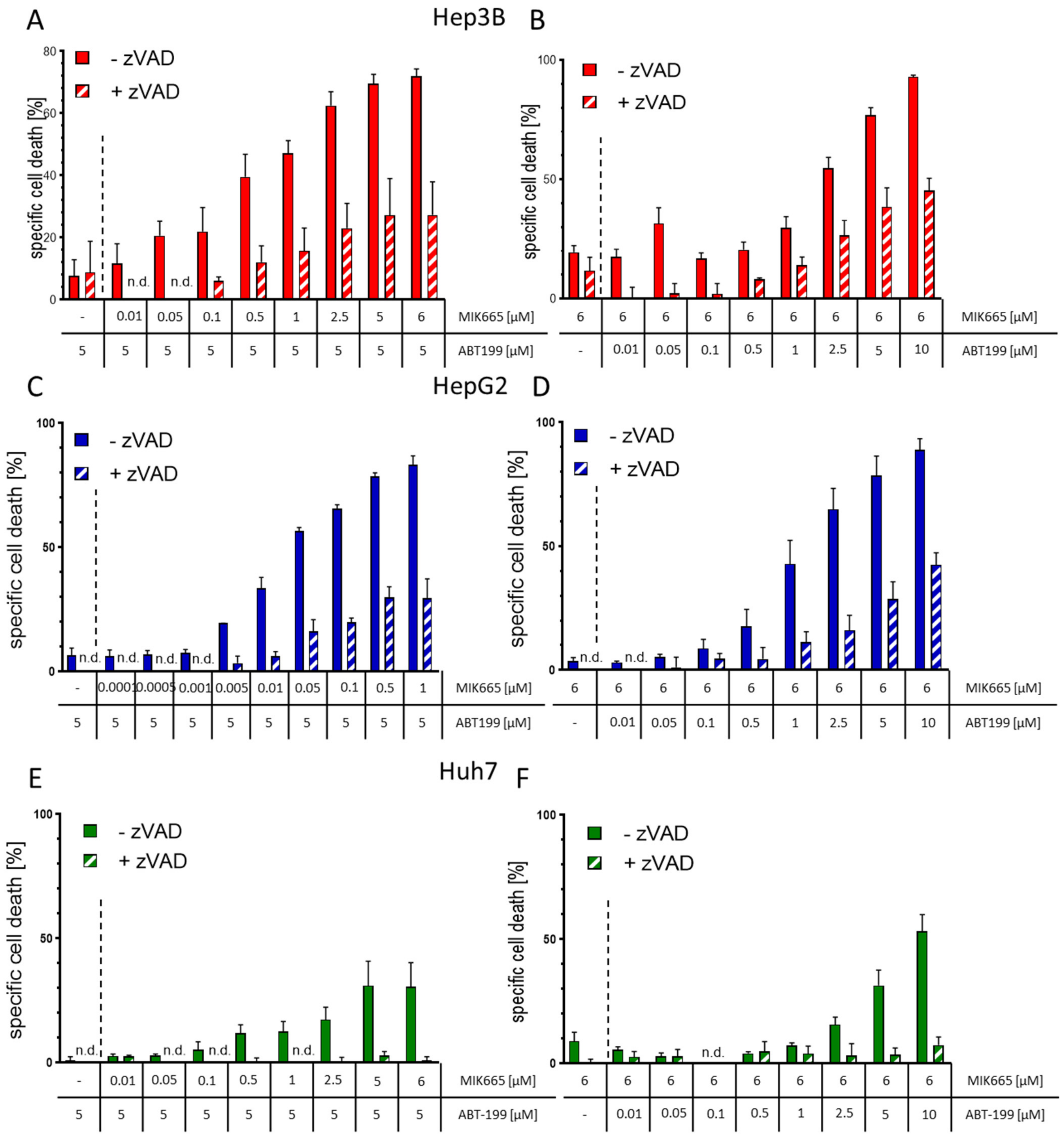

Figure S9. The pan-caspase inhibitor zVAD inhibits cell death induction by ABT-199/MIK665. Hep3B, HepG2 and Huh7 were pre-stimulated with 35 µM zVAD. After 20 min, cells were treated with BH3-mimetics for 24 h. (**A**, **C**, **E**) Cells were treated with a steady ABT-199 concentration (5 µM) and increasing MIK665 concentrations as indicated for 24 h (n=3, mean±SEM). (**B**, **D**, **F**) Cells were treated with a constant MIK665 concentration (6 µM) and increasing ABT-199 concentrations as indicated for 24 h (n=3, mean±SEM) (the dotted line separates the single treatment from the combination treatment with ABT-199 and MIK665).

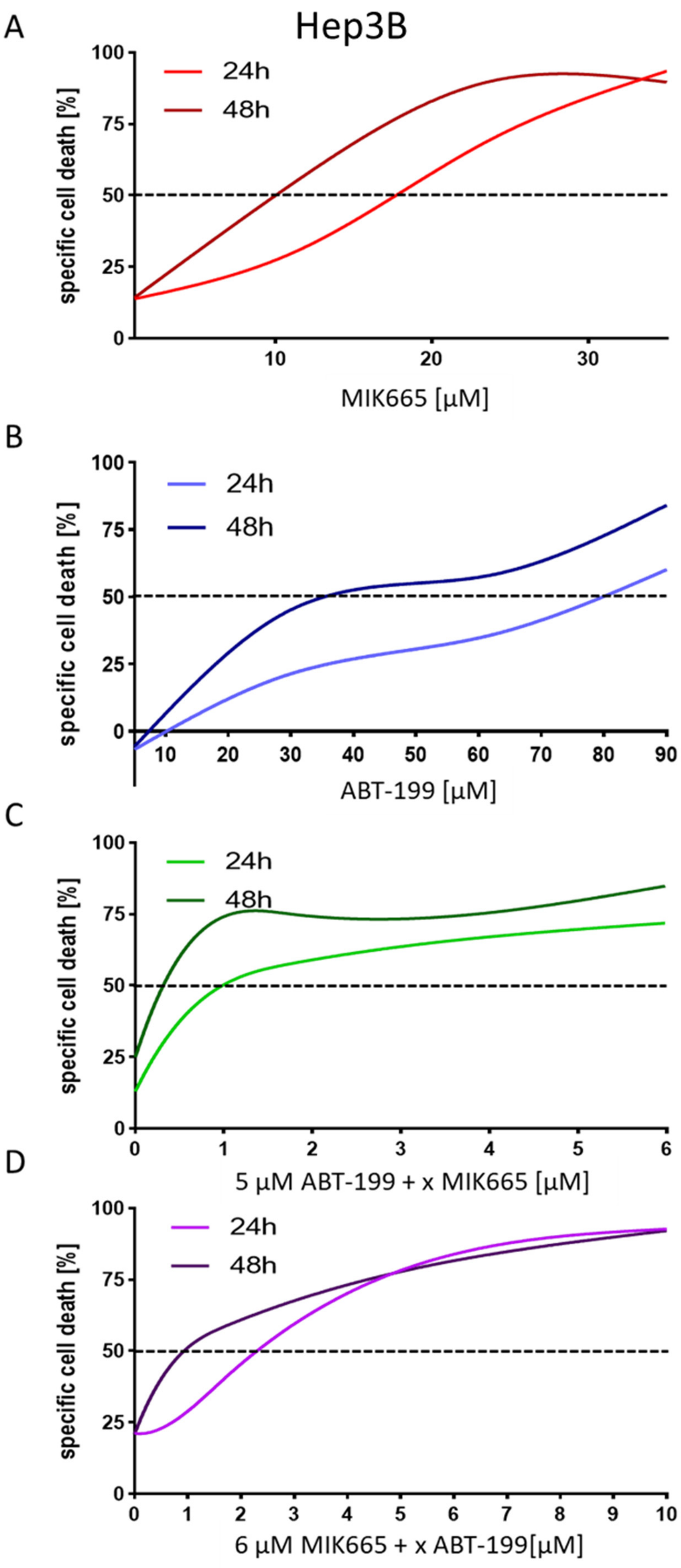

Figure S10. Combination of BH3-mimetics leads to induction of synergistic cell death in Hep3B. (**A**) Interpolation of specific cell death after 24 h and 48 h treatment with increasing MIK665 concentrations. (**B**) Interpolation of specific cell death after 24 h and 48 h treatment with increasing ABT-199 concentrations. (**C**) Interpolation of specific cell death after 24 h and 48 h treatment with 5 µM ABT-199 and increasing MIK665 concentrations. (**D**) Interpolation of specific cell death after 24 h and 48 h treatment with 6 µM MIK665 and increasing ABT-199 concentration. Calculation was executed with Graphpad Prism 8.

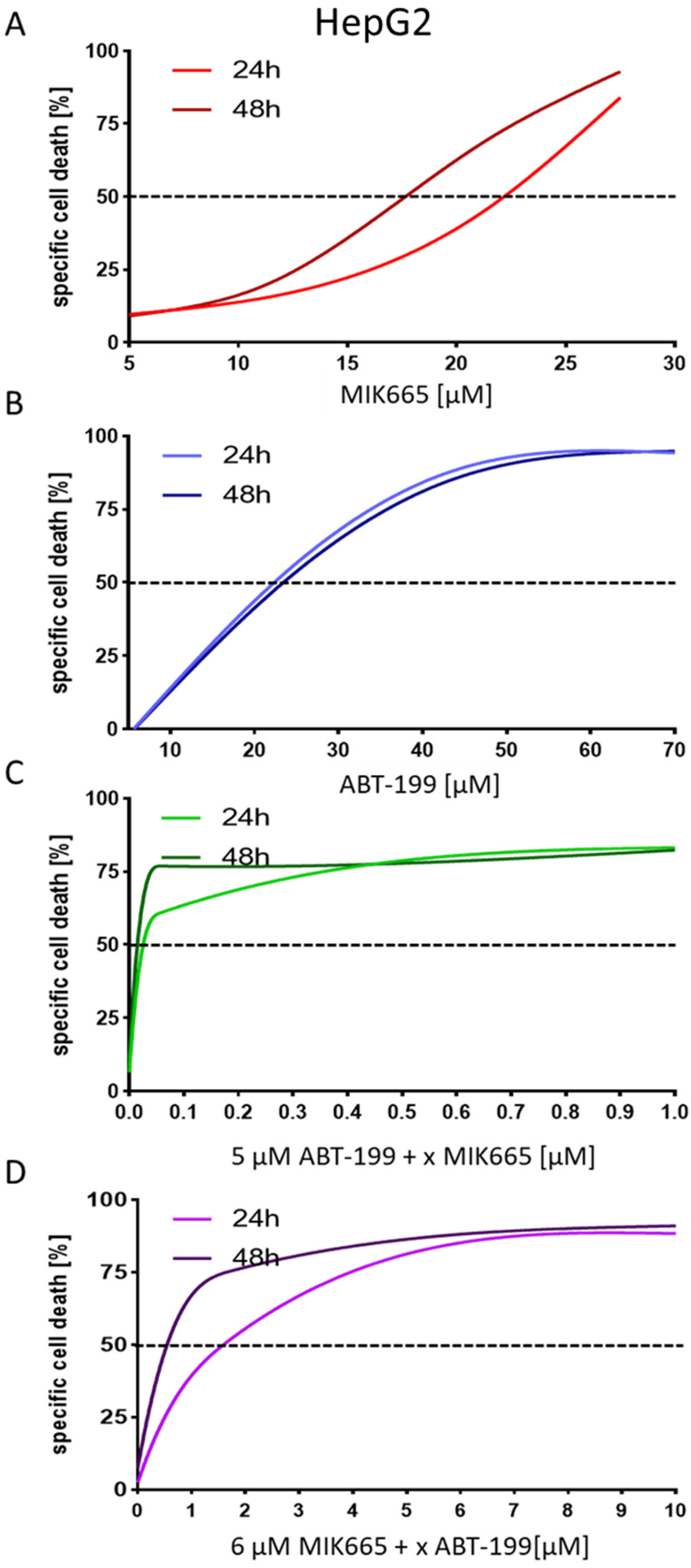

Figure S11. Combination of BH3-mimetics leads to induction of synergistic cell death in HepG2. (**A**) Interpolation of specific cell death after 24 h and 48 h treatment with increasing MIK665 concentration. (**B**) Interpolation of specific cell death after 24 h and 48 h treatment with increasing ABT-199 concentration. (**C**) Interpolation of specific cell death after 24 h and 48 h treatment with 5 µM ABT-199 and increasing MIK665 concentration. (**D**) Interpolation of specific cell death after 24 h and 48 h treatment with 6 µM MIK665 and increasing ABT-199 concentration. Calculation was executed with Graphpad Prism 8.

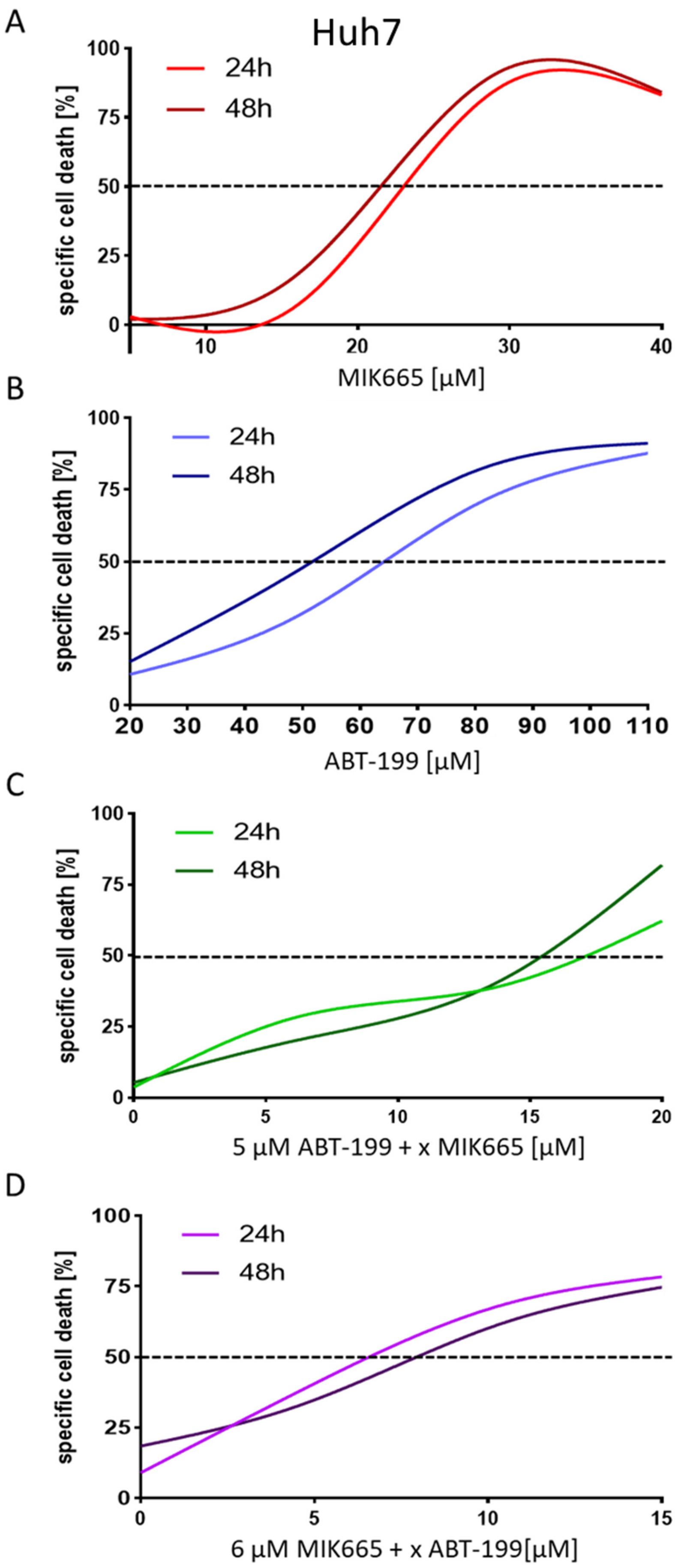

Figure S12. Combination of BH3-mimetics leads to induction of synergistic cell death in Huh7. (**A**) Interpolation of specific cell death after 24 h and 48 h treatment with increasing MIK665 concentration. (**B**) Interpolation of specific cell death after 24 h and 48 h treatment with increasing ABT-199 concentration. (**C**) Interpolation of specific cell death after 24 h and 48 h treatment with 5 μM ABT-199 and increasing MIK665 concentration. (**D**) Interpolation of specific cell death after 24 h and 48 h treatment with 6 μM MIK665 and increasing ABT-199 concentration. Calculation was executed with the Graphpad Prism 8.

Supplementary Methods

Determination of mitochondrial membrane potential: To determine mitochondrial membrane potential, cells were incubated with 30 μM Tetramethylrhodamine ethyl ester (TMRE, sigma) for 30 min. As a positive control for mitochondrial depolarization, cells were treated with 80 nM Carbonyl cyanide-p-trifluoromethoxyphenylhydrazone (FCCP) for 4 h an

d then membrane potential was determined.
